# A Platform for Assessing Cellular Contractile Function Based on Magnetic Manipulation of Magnetoresponsive Hydrogel Films

**DOI:** 10.1002/advs.202207498

**Published:** 2023-07-23

**Authors:** Moran Yadid, Mario Hagel, Megan Beldjilali Labro, Baptiste Le Roi, Carina Flaxer, Eli Flaxer, A. Ronny Barnea, Shai Tejman‐Yarden, Eric Silberman, Xin Li, Rossana Rauti, Yael Leichtmann‐Bardoogo, Hongyan Yuan, Ben M. Maoz

**Affiliations:** ^1^ The Azrieli Faculty of Medicine Bar Ilan University 8 Henrietta Szold St. Safed 1311502 Israel; ^2^ The Shmunis School of Biomedicine and Cancer Research Tel Aviv University Tel Aviv 69978 Israel; ^3^ Department of Biomedical Engineering Tel Aviv University Tel Aviv 69978 Israel; ^4^ AFEKA – Tel‐Aviv Academic College of Engineering Tel‐Aviv 69107 Israel; ^5^ The Edmond J. Safra International Congenital Heart Center Sheba Medical Center Ramat Gan 52621 Israel; ^6^ The Engineering Medical Research Lab Sheba Medical Center Ramat Gan 52621 Israel; ^7^ The Sackler School of Medicine Tel Aviv University Tel Aviv 69978 Israel; ^8^ Shenzhen Key Laboratory of Soft Mechanics and Smart Manufacturing Department of Mechanics and Aerospace Engineering Southern University of Science and Technology Shenzhen 518055 China; ^9^ Department of Biomolecular Sciences University of Urbino Carlo Bo Urbino 61029 Italy; ^10^ Sagol School of Neuroscience Tel Aviv University Tel Aviv 69978 Israel; ^11^ The Center for Nanoscience and Nanotechnology Tel Aviv University Tel Aviv 69978 Israel

**Keywords:** afterload, cardiac in vitro models, force application on cells, magnetic gels, preload

## Abstract

Despite significant advancements in in vitro cardiac modeling approaches, researchers still lack the capacity to obtain in vitro measurements of a key indicator of cardiac function: contractility, or stroke volume under specific loading conditions—defined as the pressures to which the heart is subjected prior to and during contraction. This work puts forward a platform that creates this capability, by providing a means of dynamically controlling loading conditions in vitro. This dynamic tissue loading platform consists of a thin magnetoresponsive hydrogel cantilever on which 2D engineered myocardial tissue is cultured. Exposing the cantilever to an external magnetic field—generated by positioning magnets at a controlled distance from the cantilever—causes the hydrogel film to stretch, creating tissue load. Next, cell contraction is induced through electrical stimulation, and the force of the contraction is recorded, by measuring the cantilever's deflection. Force–length‐based measurements of contractility are then derived, comparable to clinical measurements. In an illustrative application, the platform is used to measure contractility both in untreated myocardial tissue and in tissue exposed to an inotropic agent. Clear differences are observed between conditions, suggesting that the proposed platform has significant potential to provide clinically relevant measurements of contractility.

## Graphical abstract

A novel in vitro platform that enables the measurement of cardiac function's key indicator: contractility under stress. This dynamic tissue loading platform utilizes a magnetoresponsive hydrogel cantilever to apply controlled tissue load, while electrical stimulation induces cell contraction, which allows to obtain clinically relevant parameters such as pressure–volume loops.

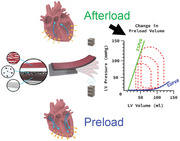

## 1 Introduction

**Figure 1:**
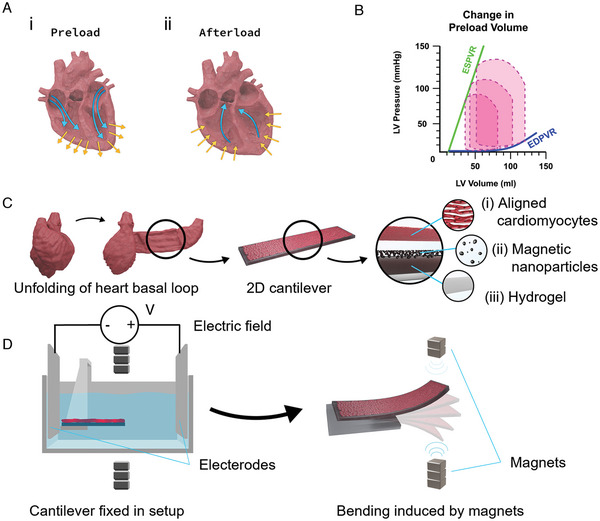
Concepts integral to the DTL setup. A) Blood flow directions, induced by pressure and opening/closing of the valves, result in (Ai) preload and in (Aii) afterload in the human heart. B) Pressure–volume loops (PVL) under varying ventricular diastolic filling volumes. The PVLs define the end diastolic pressure‐volume relationship (EDPVR) and the end systolic pressure‐volume relationship (ESPVR), which together define the passive properties and the contractility of the heart, respectively. C) The heart is unfolded into a 2D band consisting of basic anisotropic muscle layers. This basic structure can be recapitulated by engineering anisotropic cardiac tissues on soft micropatterned hydrogels. The 2D hydrogel cantilever consists of three components: (Ci) gelatin‐based hydrogel, (Cii) MNPs which are embedded in the hydrogel, and (Ciii) aligned CMs on the microgrooved surface. D) The proposed DTL setup enables loading of the engineered tissues on the hydrogel cantilever by externally applied magnetic force, using stacked magnets above and below the cantilever (depicted as dark gray squares). A pair of square platinum electrodes, shown in light gray, positioned on opposite sides across the cantilever are used to induce electrical stimulation, activating cardiomyocyte contraction.

Heart failure remains a leading cause of death worldwide^[^ ^1^ ^]^ and cardiovascular diseases place a heavy burden on the health care and drug development globally.^[^ ^2^ ^]^ Accordingly, there is an ongoing need to develop novel therapeutics and predictive preclinical models for heart disease. In vivo and whole‐organ models can be highly complex to implement and interpret, whereas simple cardiomyocyte (CM) cultures are limited in their capacity to represent various cardiac functionalities. Thus, the development of advanced in vitro models that closely mimic the physiological properties and working conditions of the heart is of particular interest. Several such models have been developed, reproducing structural and functional hallmarks of the native myocardium, using either animal or human CMs.^[^ ^3^, ^4^, ^5^, ^6^, ^7^, ^8^ ^]^ These models are built by creating an in vivo—like extracellular matrix and using substrates with biophysical cues and mechanical properties similar to those of native tissue, thereby recapitulating the native microenvironment. However, these models do not capture a key physiological feature of the heart that is crucial to the assessment of cardiac function: the dynamic loading conditions, or pressures, to which the heart is subjected before and during contraction, referred to, respectively, as preload and afterload. Preload (**Figure** **1**Ai) reflects the prestretch of the CMs against the filled ventricle, right before contraction. Afterload (Figure 1Aii), in turn, is represented by the aortic pressure against which the myocardium contracts during systole.^[^ ^9^ ^]^

More specifically, the capacity to model specific loading conditions is necessary for the measurement of contractility, physiologically defined as the ability of the heart to eject a stroke volume under given preload and afterload values.^[^ ^9^, ^10^ ^]^ Contractility is a key physiological indicator of cardiac function, as it reflects the heart's capacity to adapt cardiac output to meet the body's demand for oxygenated blood, also known as the Frank–Starling mechanism.^[^ ^11^ ^]^ In vivo, contractility is assessed at the whole‐heart level, by measuring pressure–volume relations (PVR) over a range of preload conditions, and calculating the end systolic slope of the PVR, termed Emax (maximal elastance) (Figure 1B).^[^ ^12^ ^]^ Such measurements can only be obtained in vivo or ex vivo. Notably, contractility manifests not only at the whole‐heart level but also at the single myocyte level, as loading conditions directly affect CM contraction.^[^ ^13^ ^]^ Moreover, though the heart is a 3D structure, it is composed of a cardiac sheet that can be disassembled into 2D laminar sheets^[^ ^14^, ^15^ ^]^ (Figure 1C). Together, these characteristics suggest that it should technically be possible to assess contractility in 2D in vitro models comprising CMs, by measuring contractions at the cell‐ or tissue level. Yet, existing in vitro models cannot be used to obtain such measurements, as they do not provide a means of dynamically adjusting the loading conditions.

Here, we present an in vitro platform that provides these capabilities. The core premise of our platform is to enable external magnetic forces to be used to control the loading conditions to which a functional myocardial tissue sample is exposed. To construct this dynamic tissue‐loading platform (DTL), we fabricated 2D engineered myocardial tissues on thin magnetoresponsive hydrogel cantilevers. Since the physicochemical properties of hydrogels are highly tunable, and since hydrogels have been shown to fit for engineering cellular microenvironments,^[^ ^16^, ^17^ ^]^ we sought to use gelatin as the base material for the cantilevers. Moreover, magnetic actuation of collagen hydrogels has been demonstrated for applying mechanical stretch and inducing extracellular matrix alignment.^[^ ^18^ ^]^ Therefore, we suggest that exposing a magnetoresponsive cantilever to an external magnetic field should stretch the hydrogel film too, thereby creating preload (Figure 1C,D). To assess the tissue's capacity to contract given a particular level of preload, we measure electrically induced contractions and calculate the tissue‐generated forces. The latter calculation is achieved through video analysis of the deflecting cantilevers and the integration of data into mechanical models based on the deflecting beam theory. This system can be used to derive clinically relevant preload‐dependent force–length relationships, which, we suggest, provide an in vitro parallel to the pressure‐volume relationship (PVR).

To demonstrate how the DTL might be applied to assess cardiac contractility in vitro, we derived force–length relationships for contracting CM‐tissues exposed to different loading conditions, both for untreated tissue and for tissue exposed to digoxin, an inotropic agent.^[^ ^19^, ^20^ ^]^ Using the DTL platform, we were able to detect changes in the contractility of the myocardial tissue in response to the application of the inotropic stimulus. Specifically, compared with tissue that was not exposed to digoxin, tissue exposed to digoxin was characterized by a steeper response of the generated force to changes in the preload. These results demonstrate that our in vitro system offers the capacity to mimic the loading of the heart in a dynamic manner and to measure its contractile state in response to pharmacological stimuli.

## 2 Results and Discussion

### 2.1 Fabricating Magnetoresponsive Hydrogel Cantilevers That Support Myocardial Tissue Growth

**Figure 2:**
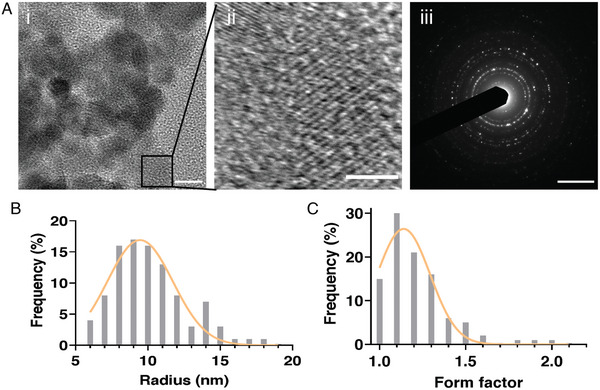
A) Characterization of magnetic nanoparticles (MNPs). (Ai) Image of MNPs under transmission electron microscopy. (Aii) Magnification of Ai, used to determine the lattice spacing, which resulted in *d* = 0.48 nm. (Aiii) Scattered electron diffraction (SAED) image showing the polycrystallinity of the MNPs. Scale bars: 10 nm, 4 nm, and 5 1 nm^−1^, respectively. B) Distribution of the measured MNPs radii. C) Distribution of the MNPs form factor measured as the ratio between the primary and secondary radii, when the MNP is considered an elliptical shape (*n* = 200).

**Figure 3:**
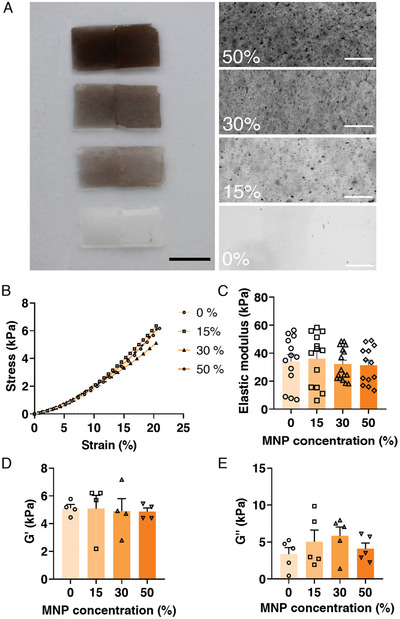
Mechanical properties of the magnetic hydrogels (Mgels). A) The left side shows Mgels with different MNP concentrations. The right side shows uniform MNP distribution in the hydrogel, identified by microscopy. Scale bars: 10 mm and 200 µm, respectively. B) Stress–strain curves obtained using a compression test, and fitting curves according to Ogden model of second order. C) Elastic modulus as estimated from the linear portion of the fitted curves. D) Storage modulus – *G*′. E) Loss modulus – *G*″. Both identified through frequency sweeps using a rheometer. One‐way ANOVA with Tukey's multiple comparison test was run for each MNP concentrations (B–E), and no statistical differences was found (*p* ≥ 0.05). Furthermore, error bars are shown as standard error of mean (SEM).

Our first step in establishing the DTL was to create magnetoresponsive hydrogel (Mgel) cantilevers that would allow anisotropic myocardial tissues to grow on their surface. To create the Mgel, we embedded iron oxide magnetic nanoparticles (MNPs) (**Figure** **2**) in a gelatin‐based hydrogel (**Figure** **3**), which was then crosslinked enzymatically to form covalent bonds and stabilize the hydrogel. After characterizing the mechanical properties of the Mgel material and verifying its amenability to cell growth, we engineered myocardial tissues on the surface of Mgel cantilevers by micropatterning and crosslinking the gel prior to seeding CMs; the micropatterns ensured that CMs would be aligned in a manner mimicking the myocardial tissue structure (Figure S1, Supporting Information). The results of these procedures are summarized in what follows.

#### 2.1.1 Mgel Fabrication and Characterization

Water‐soluble MNPs were prepared according to Fried et al.^[^ ^21^ ^]^ The procedure resulted in polycrystalline MNPs (Figure 2A) with fairly circular shape, and an average radius of 10 ± 0.26 nm (Figure 2B), and a form factor of 1.2 ± 0.2 (Figure 2C). While introducing nanoparticles into hydrogels may modify their mechanical properties, it has been demonstrated that nanoparticles in this size range, ≈10 nm or below, have a negligible effect on the hydrogel's mechanical properties.^[^ ^22^, ^23^ ^]^ Moreover, particles with diameters above 20 nm tend to have irregularity and polydispersity.^[^ ^24^ ^]^ Therefore, the MNPs that were produced here are expected to be uniform and minimally affect the mechanical properties of the hydrogel. Moreover, the shape and form factor of MNPs are also known to affect their properties, and effects on the hydrogel composites.^[^ ^25^, ^26^, ^27^ ^]^ While anisotropic nanoparticles are known to enhance hydrogel's mechanical properties, the isotropy of small, spherical nanoparticles is not expected to prominently modify the composite material properties.^[^ ^26^ ^]^ Here, a form factor of 1.2 indicates a typical nanosphere shape, with a small diameter of ≈10 nm, suggesting that the fabricated MNPs should not significantly alter the mechanical properties of the hydrogel, and are uniformly dispersed within it.

The MNPs were then mixed with gelatin in different concentrations (MNP relative concentrations of 15%, 30%, and 50%) to create a magnetoresponsive gel (Movie S1i, Supporting Information) in which the MNPs were uniformly embedded (Figure 3A; see the Experimental Section for more information). This uniform distribution of the MNPs within the hydrogel should enable an equal response to the magnetic field across the whole cantilever. The gelatin concentration (20%) was set to produce hydrogels with stiffness values approximately matching those of the native heart matrix, ≈10–15 KPa (Figure 3).^[^ ^6^, ^28^ ^]^

We then compared the mechanical properties of hydrogel samples containing MNPs to the properties of pristine gelatin hydrogels, to verify that the former would be amenable to cardiac tissue growth. Stress–strain curves were derived from a uniaxial compression test and fitted according to an Ogden model referring to the hydrogel as a second‐order neo‐Hookean material. The stress–strain curves did not differ substantially between pristine gelatin hydrogels and hydrogels containing MNPs at different concentrations (Figure 3B). Next, the elastic modulus was estimated from the slope of the stress–strain curves in the linear deformation range^[^ ^29^, ^30^ ^]^ (Figure 3C). The elastic modulus values obtained for the gels with different MNP concentrations were not significantly different from one another or from those of pristine gelatin hydrogel and were well within the range matching the stiffness of the native myocardial matrix.^[^ ^6^, ^31^, ^32^ ^]^ Finally, rheological measurements were performed to evaluate the hydrogels’ storage modulus (Figure 3D) and loss modulus (Figure 3E). These measurements demonstrated that the presence of MNPs at different concentrations did not significantly alter the elastic and viscous properties of pristine gelatin hydrogel.

To create the films that would serve as cantilevers in our platform, we cast the gels into custom molds (Figure S1, Supporting Information) with dimensions of 20 mm × 10 mm and a thickness of 200 µm. This thickness was chosen to ensure that the cantilevers would be thin enough to deflect in response to CM contraction on the one hand, and thick enough to respond to an external magnetic field on the other (Movies S1i and S2, Supporting Information). As will be elaborated in subsequent sections, our cantilever is constructed by clamping half the film to a surface while the other half is free to move. In what follows, for convenience, we occasionally use the term “cantilever” to refer not only to this assembly but also to the rectangular film itself.

Notably, the magnetoresponsive hydrogel cantilevers are expected to remain stable during the time course of the experiments, and even longer, as previously demonstrated for hydrogels composed of 10% gelatin crosslinked with 4% transglutaminase^[^ ^33^ ^]^ Moreover, Goudu et al. have shown that a similar magnetoresponsive hydrogel based on 10% methacrylated gelatin, completely degrade after 8 days when incubated in a solution with a matrix metalloproteinase‐2 (MMP‐2) concentration of 3.3 µg mL^−1^, which is ≈12‐fold higher than in typical biological environment.^[^ ^34^ ^]^ Therefore, it can be expected that a time window of at least two weeks can be allowed for future applications.

#### 2.1.2 Engineering Cardiac Tissues on the Surface of Mgel Cantilevers

**Figure 4:**
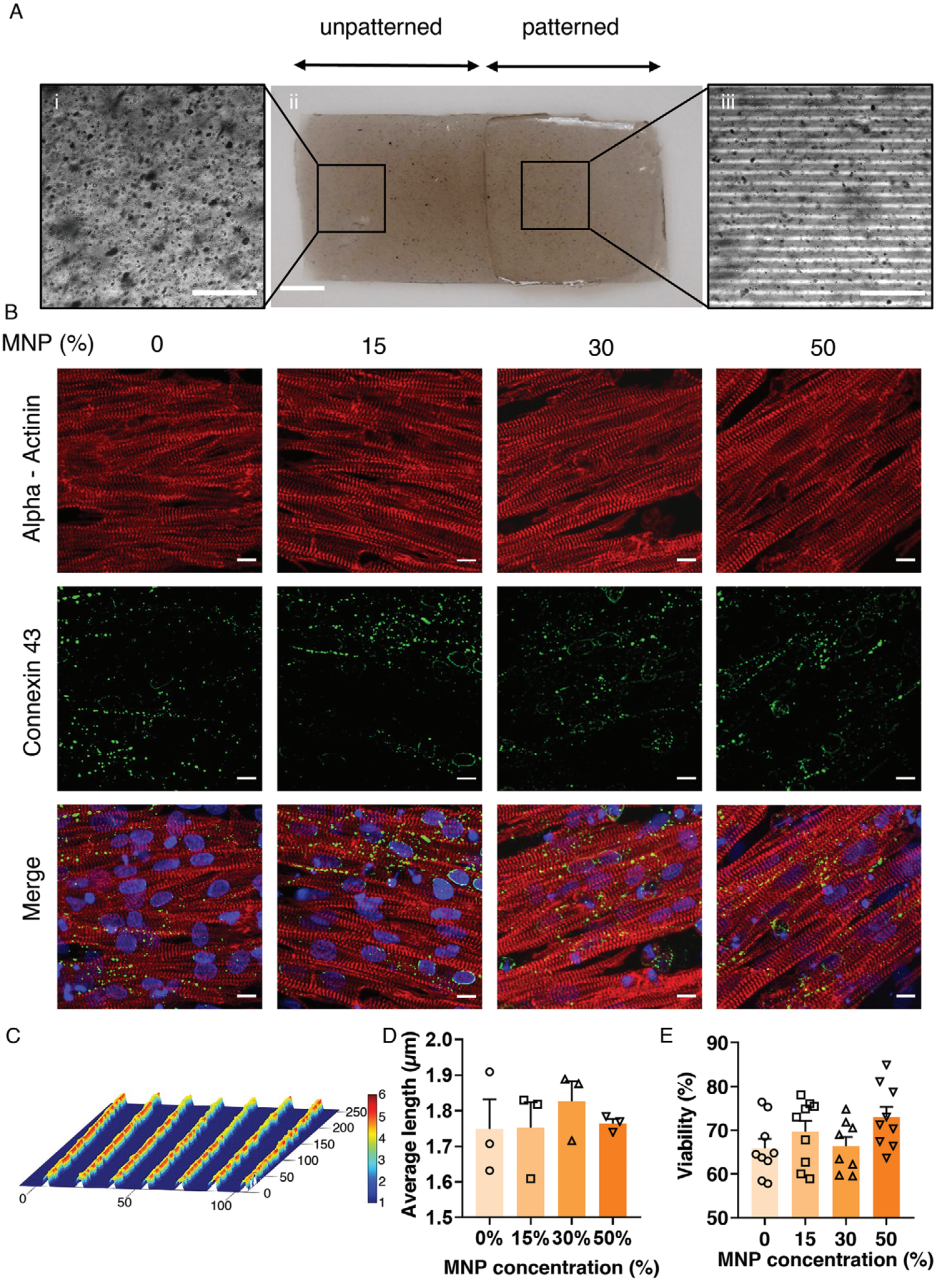
A) Characterization of the patterned hydrogel cantilevers, immunostaining, cell viability, and orientation order parameter. (Ai) A magnified area of the unpatterned side of a cantilever with 15% MNPs (Aii) An entire cantilever is shown with the unpatterned on the left and micropatterned area with the aligned lines pattern on the right. (Aiii) An enlarged image of the micropatterned cantilever part. Scale bars: 100, 250, and 100 µm, respectively. B) Immunostaining of cardiomyocytes on hydrogels with different MNP concentrations. DAPI – Nucleoli (blue). Alpha‐actinin – sarcomeres (red). Connexin 43 – tight junctions (green). The different columns show immunostained samples from the various MNP concentrations (0–50%). Scale bars: 10 µm. C) Ridges and grooves topography of the patterned hydrogel, as derived by a profilometer. Scale bar in µm. D) Sarcomere length measured for CM on different MNP concentrations. Five samples were taken from three separate images for each concentration and averaged. E) % viability on hydrogels with different MNP concentrations, as assessed using a live/dead assay. One‐way ANOVA with Tukey's multiple comparison test was run (D, E) and no statistical differences was found (*p* ≥ 0.05). Error bars are shown as standard error of mean (SEM).

Our next step was to engineer laminar, anisotropic cardiac tissues, mimicking the structure of a myocardial sheet,^[^ ^35^, ^36^ ^]^ on the surface of the Mgel cantilevers. This was achieved by micromolding microgrooves (**Figure** **4**A) (10 µm wide, 5 µm deep grooves, separated by 5 µm wide ridges) on the surface of the cantilevers, onto which we subsequently seeded CMs (neonatal rat ventricular myocytes); the purpose of the microgrooves was to induce alignment of the CMs to recapitulate the morphology of myocardial tissue^[^ ^7^, ^8^, ^25^ ^]^ (Figure 4B–D; Figure S2, Supporting Information).

To assess whether the micropatterned Mgels support CM viability, alignment, and functionality, we seeded CMs on cantilevers with different properties—namely, patterned and unpatterned cantilevers containing MNPs at different concentrations (ranging from 0 to 50%)—and, 5 days later, we examined the formed CM‐tissues.

First, we stained the myocytes to verify that they exhibited the prevalent cardiac sarcomere proteins α‐actinin and connexin‐43, in the expected morphology after 5 days in culture (Figure 4B). Furthermore, the sarcomere length was measured from five samples taken from three images for each concentration and the length was averaged to ensure normal morphological expression (Figure 4D). At this time point, we assessed CM viability by performing a live/dead assay using propidium iodide and Hoechst staining (Figure 4E). MNP concentration did not affect cell viability.

Next we examined the extent to which the micropatterning of the Mgels indeed induced cell alignment, mimicking tissue morphology. To this end, we compared CM‐tissues grown on patterned versus unpatterned cantilevers and showed that cantilevers patterned with microgrooves exhibited anisotropic tissues, whereas unpatterned cantilevers were less amenable to tissue adhesion and did not promote tissue alignment (Figure S2, Supporting Information). As is apparent from the α‐actinin images (Figure 4B), the formation of anisotropic tissues was facilitated both on pristine hydrogel cantilevers and on the MNP‐embedded cantilevers.

Finally, we used calcium imaging to assess whether CMs retained their contractile function when cultured on Mgel. The measured calcium transients demonstrated regular calcium waves and propagation through the tissue, indicating that the CMs contract spontaneously (Figure S3, Supporting Information)

Together, these data indicate that our micropatterned Mgel cantilevers support viable and functional cardiomyocytes, which grow in an aligned structure matching myocardial tissue morphology.

### 2.2 Construction and Characterization of the DTL System in the Absence of Cells

#### 2.2.1 Setup of the DTL System

**Figure 5:**
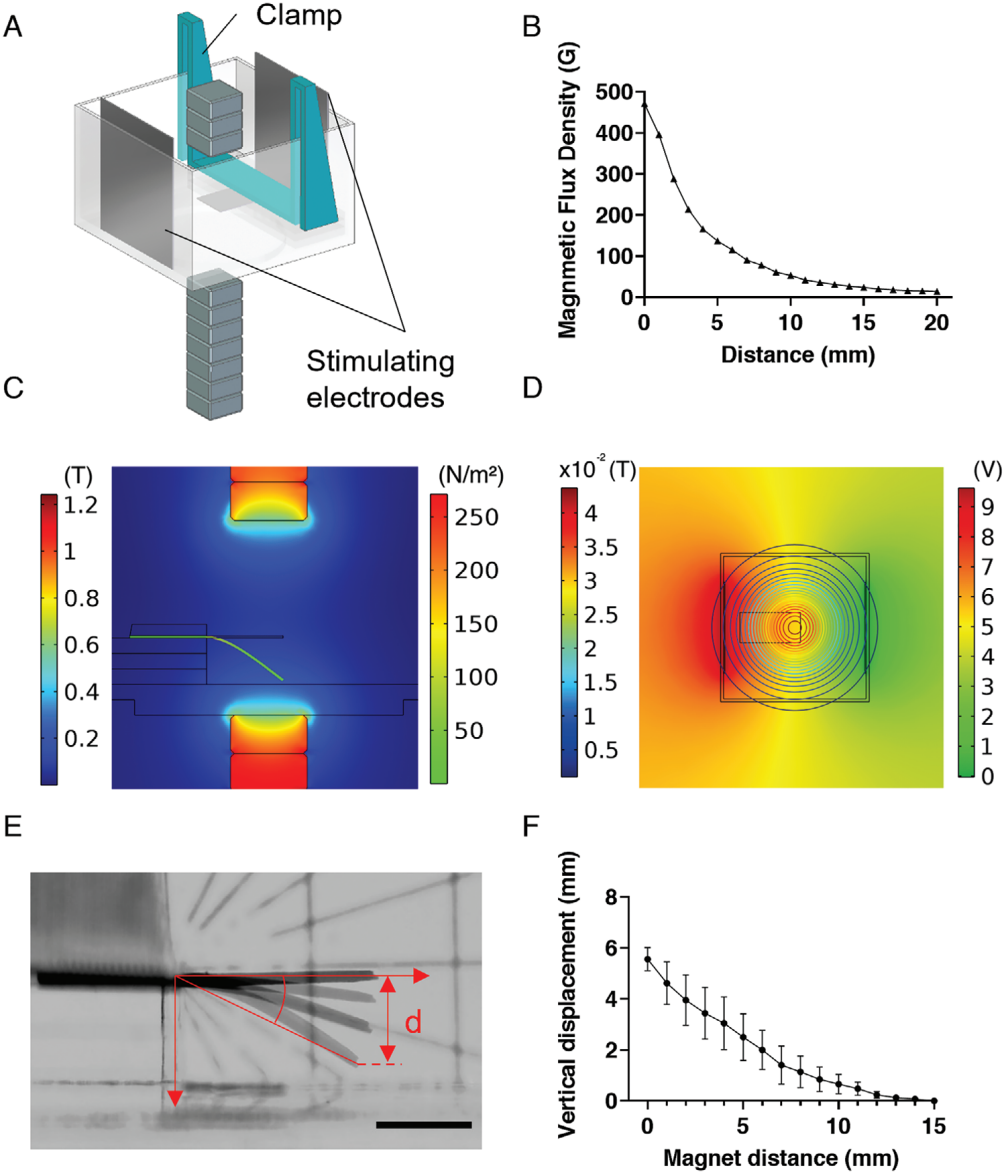
Analysis of cantilever's bending. A) CAD model used for the simulations. B) Magnetic flux density as a function of distance from the magnets. Values were measured using seven magnets, as used in the experimental setup (each 10 mm × 10 mm × 5 mm; magnetic flux: ≈1.2 T), mounted on the lower motor. C) Magnetic flux density distribution at the sagittal plane section of the setup and stress distribution in the cantilever, as calculated using a COMSOL Multiphysics simulation. Left color bar: magnet flux density in Tesla. Right color bar: von Mises stress applied on cantilever in N m^−^^2^. D) Top view of the applied electrical field on the cantilever during electrical stimulation phase. Left scale bar, magnetic flux density as presented in contour lines, in Tesla. Right color bar, electrical potential in V. E) Cantilever bending in the setup, as the magnet is positioned at various distances from the chamber's floor. *α* represents the angle of displacement and *d* the vertical displacement. F) Vertical displacement of the cantilever, *d*, as a function of the magnets’ distance from the bottom of the DTL chamber. When the magnet is at 0 distance from the chamber's bottom, it is 8 mm away from the plane of the cantilever.

The next step was to construct our DTL system. Recall that this system aims to fulfill these three main functionalities: i) provide a means of controlling the loading conditions to which myocardial tissue is subjected; ii) induce CM contraction under given loading conditions; and iii) provide a means of measuring contractility. Thus, the DTL system is composed of three main parts, each corresponding to one of these functionalities: i) magnetic control, which enables the user to manipulate the strain of the Mgel cantilever, and thus induce preload on the plated CM‐tissue (Figure 1D); ii) electrical stimulation—which initiates CM contraction (**Figure** **5**A), and iii) a camera—which enables the deflection of the cantilever to be monitored at a high frame rate (240 fps), to facilitate extraction of clinically relevant parameters from the contracting tissue (e.g., stress–strain curves) (Figure S4, Supporting Information). The electrical stimulation also acts as a trigger to correctly time the application of the magnetic field prior to electrical activation of the tissue, therefore setting the preload.

The setup of the DTL is presented in schematic form in Figure 1D. Additional details are presented in the Experimental Section (DTL Setup: Platform Overview) and Figure S4 of the Supporting Information. Briefly, the system consists of a clear chamber (47 mm × 47 mm × 30 mm) made of polymethyl methacrylate (PMMA); the chamber is filled with Dulbecco's phosphate buffered saline (PBS), in which a Mgel cantilever is positioned on top of three spacers (each: 45 mm × 15 mm × 2 mm), centered and normal to their long side. The cantilever is constructed such that half of the length (10 mm) of the Mgel film is fastened using a PLA (polylactic acid) clamp, which is placed on top of it and fixed between the walls of the PMMA (poly(methyl methacrylate) chamber. The remaining portion (10 mm × 10 mm of the Mgel) hovers in the medium, 6 mm above the bottom of the inside of the chamber. Three magnets, with each 10 mm × 10 mm × 5 mm dimension and ≈1.2 T magnetic flux are positioned above the chamber at a distance of 15 mL from the plane of the cantilever, and 7 magnets are stacked on a motor arm below the chamber (Figure 5A). The motor arm can be moved to adjust the distance of the magnets from the chamber, thereby determining the level of magnetic force to which the cantilever is exposed. The upper magnets are used to calibrate Mgel film in a horizontal initial position, due to gravity, and are not moved during the experiment. The magnets below are used to prestretch the Mgel cantilever and the motor arm can be moved up to 10 mm close, relative to the initial, horizontal Mgel position. In addition, two platinum electrodes are positioned on opposing sides of the chamber, perpendicular to the long axis of the Mgel film, while it lays between them (Figure 1D). The electrodes were fixed onto the chamber walls using clamps. The electrodes are plugged into an amplifier, which is connected to a computer, where the applied voltage and current can be set and adjusted. Electrical stimulation is used to initialize deliberated cardiomyocyte contractions. Due to the relatively high buffer volume in the experimental chamber, it is expected that relatively high voltages would be needed to activate the tissue. Therefore, to reduce hydrolyzation of the aqueous media, platinum was selected as the electrode material due to its biocompatibility and low reactivity.^[^ ^37^, ^38^ ^]^

#### 2.2.2 Characterization of Magnetic Fields and Measurement of Cantilever Deflection in Response to External Magnetic Force

After establishing our setup, we characterized the range of magnetic flux that could be applied (Figure 5B), and the response of the Mgel cantilever (in the absence of cells) to the magnetic field (Figure 5C–F; Figure S5 and Movie S1i, Supporting Information). As expected, the magnetic field decayed as a function of the cubic distance from the magnets (*r* ^3^). This meant that the magnet had a negligible effect on the deformation of the Mgel cantilever when it was positioned 20 mm away from the magnets.

To further characterize the system, and to ensure that the entire cantilever was exposed to a homogeneous field, we used a finite‐element simulation to simulate the magnetic flux density at the plane of the cantilever, and the distribution of the electric field between the platinum electrodes (Figure 5C,D; Movie S2, Supporting Information). As Figure 5C,D shows, both the magnetic and the electric fields were homogenous around the tip (and center) of the Mgel cantilever.

**Figure 6:**
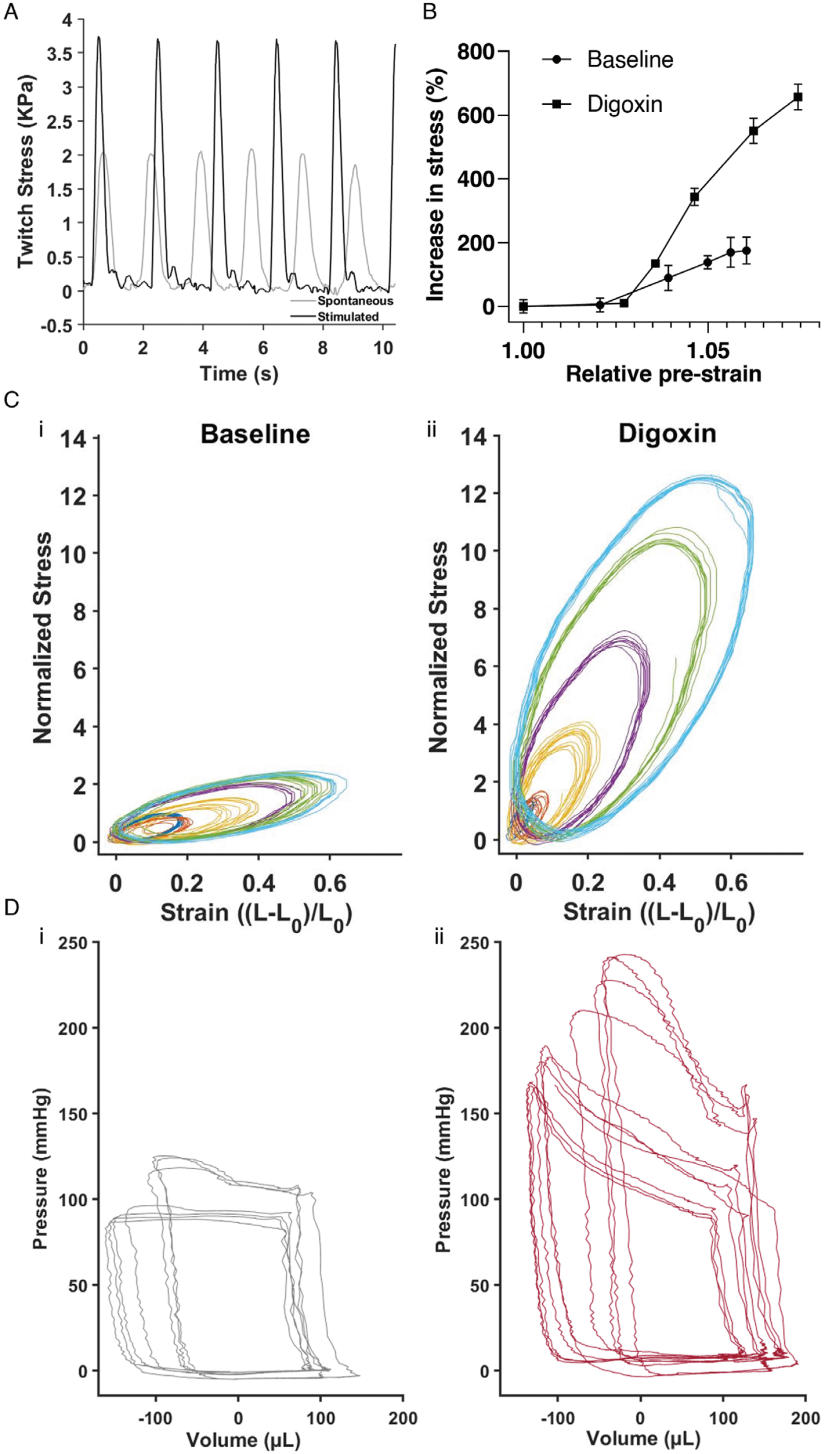
Measurements of contractile function using the Mgel and DTL platform. A) Force of spontaneous and electrically stimulated contractions over time. B) 2D equivalent of the PV loops–Normalized stress–strain loops for (Bi) untreated CM‐tissue and (Bii) CM‐tissue exposed to digoxin. C) Stress–length relationships for untreated tissue and for tissue exposed to digoxin, which exhibit steeper relationship. D) Pressure–volume loops (Di) without and (Dii) with 0.5 mg kg^−1^. Digoxin, derived from a rat in vivo while varying the load.

Next, we examined the deflection of the Mgel cantilever in response to externally applied magnetic field. The vertical displacement of the cantilever's tip and its angle were measured as a function of the magnets’ distance from the plane of the cantilever (Figure 5E,F; Figure S5A, and Movie S1i, Supporting Information), demonstrating monotonically decreasing relations. We subsequently used the beam model to translate the measured vertical displacement of the cantilever's tip into tension force generated by the cells, corresponding to the magnetic force applied (Figure 6; Equation (3)). When we plotted the vertical displacement against the angle of deflection, a linear relationship was obtained (Figure S5B, Supporting Information), justifying a working hypothesis of small angle approximation—in which $\alpha =\frac{d}{L}$, where *a* is the angle of displacement relative to the resting position, *d* is its vertical displacement, and *L* the cantilever's length—and the use of the beam model.

Moreover, we measured the relaxation time of the Mgel cantilever following expulsion of the magnets (the time it takes for the cantilever to return to its initial horizontal position after the magnets are brought back to their initial, distant position) as a function of the bottom magnets’ distance from the chamber (Figure S5C,D, Supporting Information). The relaxation time monotonically increased as the magnets were positioned closer to the cantilever and was up to 410 ms when the distance between the magnets and the bottom of the chamber was zero. In experimental applications, this value has to be considered as a limiting factor when determining the frequency at which the tissue is electrically stimulated to contract (i.e., the tissue's pacing frequency).

Notably, the values of the cantilever's level of deflection in the presence of a magnetic field, as well as its relaxation time, are dependent on specific system parameters (e.g., the concentration of MNPs in the Mgel, cantilever dimensions, number of magnets stacked on the motor arm, etc.). However, once a final configuration for the platform is selected, the various parameters remain constant, whereas only the distance of the magnets is adjusted—thereby providing control over the level of prestretch to which the cantilever is subjected.

### 2.3 Using the DTL to Measure Contractility in CM‐Tissues (Untreated or in the Presence of Digoxin)

We used our platform to measure contractility in CM‐tissues in vitro, by i) inducing contraction with electrical stimulation, ii) exposing the tissues to preload using the magnetic control system, and iii) obtaining stress–strain curves under varying preload conditions as a measurement of cells’ contractile state. The latter measurements are expected to be comparable to the PVR loops, which are considered as a standard assessment of contractility.^[^ ^39^, ^40^ ^]^ We measured contractility both in untreated CM‐tissue and in tissue exposed to digoxin, an inotropic agent. In these experiments, we used cantilevers with MNP concentrations of 50% (as described in the Experimental Section).

First, we obtained baseline measurements of the spontaneous contractile activity of CM‐tissue, in the absence of external magnetic force. To this end, a cantilever with anisotropic CM‐tissue was mounted onto the platform, in a physiological buffer at room temperature, and the spontaneously occurring deflection of the cantilever, due to cell contraction, was recorded. Then, we electrically stimulated the CM‐tissue using pulses of 12 V, administered at 0.5 Hz frequency, and contractions in response to electrical stimulation were recorded. Since the cell layer was constructed on the top side of the Mgel cantilever, myocytes contraction resulted in upward bending of the film. The stress values corresponding to the recorded contractions were calculated by applying the beam model (Equation (4), **Figure** **6**A). The recorded stress levels were in accordance with previously published muscular thin film models.^[^ ^6^, ^7^, ^35^ ^]^ Stress levels generated in response to electrical stimulation (and in the absence of a magnetic field) were considered as baseline contractions with no applied preload (Movie S1ii, Supporting Information).

Next, we proceeded to measure contractions in the presence of different preload values. To generate preload, we programmed the bottom motor arm of our system, containing magnets, to move to predetermined positions relative to the bottom of the chamber, exposing the cantilever to controlled magnetic fields. Each exposure induced an initial downward deflection of the cantilever—i.e., preload—the value of which was dependent on the distance of the bottom magnets from the cantilever (Figure S5C,D, Supporting Information), where a shorter distance corresponded to higher preload. We held the cantilever in a preloaded state for 500 ms, and then, upon release of the magnets back to their baseline position, an electrical stimulation was initiated, to induce myocyte contraction (Figure S5E, Movie S1iii, Supporting Information). As described in detail in the Experimental Section (Force–Length Loops as an Equivalent Model to Pressure–Volume Loops), from the deflection of the cantilever we derived dynamic measurements of the stress and the strain of the 2D myocardial tissues, corresponding to preload and contraction. We derived quasi‐static force–length (FL) relationships from the percentage increase in average peak active force presented as a function of the relative preload (Figure 6B). Notably, this type of measurement is commonly used to assess cardiac muscle function in vivo and in vitro in muscle strips, while here as well, a steeper curve reflects enhanced contractile state.^[^ ^41^, ^42^, ^43^, ^44^, ^45^ ^]^

The dynamic stress was normalized to the peak baseline (no preload) stress and plotted against the dynamic strain for the various tested preloads (Figure 6Ci). As expected, the generated contractile forces were preload‐dependent, and increased with increased cantilever pre‐stretch (preload). As noted above, we suggest that these measurements are comparable to the whole heart measurements of PVR loops, such that they reflect an aspect of the physiological Frank–Starling regulation of contraction.

Next, we added 3 µm digoxin, a positive inotropic agent, to the physiological buffer, and 10 min later repeated the procedure described above, stimulating and recording contractions of the cantilever under varying preloads (Figure 6Cii). As in our measurements for untreated CM‐tissue, the derived stress–strain curves consistently demonstrated preload dependence. In this case, however, the preload dependence was steeper, such that the same increases in preload elicited larger increases in contraction force. The steeper preload dependency reflects the positive inotropic effect of digoxin and the increased tissue contractility. Our data show length dependence in the absence of drug exposure and a steeper length dependence under the influence of an inotropic drug, reflecting the increased muscle contractility. To validate our system, we compared the obtained response to digoxin in the DTL systems with the in vivo response, by measuring ventricular pressure and volume in a living rat. Baseline pressure and volume were recorded using a Millar catheter inserted in the left ventricle of an anaesthetized rat during stable, uninterrupted cardiac cycles (Figure S7i, Supporting Information), followed by recordings during vena cava occlusions, where the preload is changed (Figure 6Di). Expectedly, occlusions resulted in increased peak systolic pressures, associated with the changes in diastolic filling. Then, 0.5 mg kg^−1^ digoxin was injected IV, and the same procedure was repeated (Figure S7ii, Supporting Information; Figure 6Dii). Here too, preload dependent peak systolic pressures are observed. However, it is evident that following digoxin administration the preload dependent increase in pressure was steeper, similar to our observation using the DTL. Although we observe similar trends, the results are not fully comparable due to multiple reasons. Pressure–volume (PV) loops derived in vivo have a quadrilateral‐like shape due to existence of valves, while the loops obtained in the 2D DTL system are elliptical and represent solely the muscle properties. Moreover, the in vivo data represents the overall response of the animal that stems not only from the direct myocardial response to digoxin, but also from the response of the nervous system and the entire circulatory system. This issue presents an advantage of our suggested system, where we observe changes in contractile activity, due to the effect of drug on the cardiomyocytes alone, but yet obtain information that could not otherwise be obtained in traditional cellular models.

Notably, to measure the load‐independent contractility in vivo, it is required to insert a pressure–volume catheter into the left ventricle. Then, to modify the preload, the inferior vena cava (leading the returning blood into the left ventricle) is occluded to several degrees, while the cyclic changes in pressure and volume due to cardiac contraction are measured. Although this measurement faithfully represents the contractile function of the heart, it is invasive and cannot be implemented clinically in humans. Therefore, contractility measurements in humans are elusive and unattainable. Clinical assessment of the myocardial function of the human heart is mostly based on echocardiography, providing only partial information since these measurements are load dependent. Our DTL platform enables load independent assessment of myocardial contractility in 2D engineered tissues, which can be constructed from both animal cardiomyocytes and human‐derived cardiomyocytes.^[^ ^7^, ^8^, ^35^, ^36^ ^]^

## 3 Conclusion

In this work, we introduced the DTL, an in vitro experimental platform that provides a means of dynamically adjusting loading conditions on CM‐tissues, and of assessing the tissue's contractile function under these conditions—referred to as contractility. As discussed above, current in vitro platforms cannot be used to obtain such measurements, as they do not provide the capacity to control loading conditions, as can be performed in vivo or ex vivo, in animal models. Our system is based on the use of 2D engineered CM‐tissue cultured on magnetoresponsive cantilever films, which deflect in response to external (controlled) magnetic force—thereby stretching the tissue and creating preload. Although this system does not fully recapitulate the in vivo conditions, as it lacks the complexity of the 3D heart, it does not contain valves that allow pressure buildup, and does not include the neurohumoral regulation of heart function, we suggest that it can be used to assess myocardial function. Despite the shortcoming of the described DTL, we were able to use the system to derive load independent measurements of contractility in untreated CM‐tissue and in tissue exposed to an inotropic agent. The DTL platform can potentially enable changes in the afterload as well, by either setting the hydrostatic pressure in the chamber or by changing the distance of the magnets above the cantilever, which remains constant throughout the contractile cycle. Further work can focus on precisely controlling the loads in terms of force, assessment of passive tension, and real time measurements of the sarcomere length in the engineered CM‐tissue. This will enable a better definition of the loading conditions, reliable comparison between samples, and contractility assessment. This work thus constitutes a crucial step toward assessing myocardial tissue function in engineered tissues, in vitro. Notably, potential applications of the DTL are not limited to cardiac research; the platform is modular and can be used for other contractile tissues to model physiology and disease states, and to derive clinically relevant data.

## 4 Experimental Section

### Mgel Cantilever Fabrication and Characterization: Magnetic Nanoparticle Synthesis

MNPs were synthesized as previously described by Fried et al.^[^ ^21^ ^]^ Briefly, MNPs were synthesized by mixing FeCl_2_·4H_2_O:FeCl_3_ at a 1:2 ratio in a basic solution of hydroxide ammonium. This reaction was performed under nitrogen atmosphere at 85 °C for 2 h. Then, the formed magnetite Fe_3_O_4_ nanoparticles were washed several times by magnetic decantation. Following that, a hydrophilic coating of meso‐2,3‐dimercaptosuccinic acid (DMSA) was formed.^[^ ^46^ ^]^ To do so, the MNP mixture was diluted into 10 mL of hexane (Biolab 110‐54‐3, 88.198 g mol^−1^, Israel), and 50 mL of double distilled water. Once the MNPs were homogeneously suspended, the suspension was combined with aqueous DMSA 10%(M/V) (Alfa Aesar A17909, 182.22 g mol^−1^, Israel) and 20 mL of acetone that functions as an intermediate solvent of the MNP. The solution was stirred at a high frequency for 48 h at room temperature. Then the hydrophilic soluble MNPs were separated from aggregation.

### Magnetic Nanoparticle Characterization

The MNPs were characterized using transmission electron microscopy (TEM). To prepare samples for imaging, 1 mL of the MNP solution ×1000 was diluted and a drop of the diluted MNP solution was mounted on a TEM grid (Carbon Type A). The samples were then left to dry at room temperature for 48 h.

The samples were then imaged using transmission electron microscopes (DMi8, Leica, Germany; and JEM‐2010F, JEOL, Japan, equipped with a UHR pole piece), operated at 200 kV. Bright field diffraction‐ and phase‐contrast images were recorded on a K2 Summit direct electron detector (Gatan‐Ametek, USA), attached to the microscope, and set to linear mode. The morphology and distribution of the MNPs were quantified using ImageJ.^[^ ^47^ ^]^

### Preparation of Mgel Solution and Fabrication of Microgrooved Mgel Thin Films

The preparation of the Mgel solution and of the corresponding thin films was based on a previously published technique.^[^ ^6^ ^]^ Briefly, 20% w/v gelatin solution was prepared by dissolving 200 mg gelatin (Gelatin Type A, 175 bloom from porcine skin, Sigma‐Aldrich, Wisconsin, USA) in 1 mL PBS or 1 mL of PBS–MNP solution mixture, as described in **Table** **1**. The solution was vortexed and placed in a 65 °C water bath until completely dissolved. Meanwhile, 8% w/v Activa RM transglutaminase solution was prepared by dissolving 80 mg transglutaminase powder (Activa RM transglutaminase, Ajinomoto Corp, Japan) in 1 mL PBS. The solution was vortexed and placed in a 37 °C water bath until the transglutaminase was dissolved.

**Table 1:** Quantities for preparation of Mgel solution at different concentrations of MNPs

Mgel solution [%]	MNP solution [mL]	PBS [mL]
*n*	*n*	1–*n*
0	0	1
15	0.3	0.7
30	0.6	0.4
50	1	0

Note: The table shows the volume of PBS required to dilute 200 mg (10% w/v in total volume) ogelatin to receive the corresponding Mgel solution before mixing with diluted transglutaminase solution.

To prepare the Mgel cantilevers, the gelatin, and the transglutaminase solutions were thoroughly mixed using a pipette, and cast into 3D‐printed PLA (RaisePro, Raise Technologies, Inc.) molds clamped on a glass cover slide (Figure S1A, Supporting Information). Next, 40 µL of the mixture was dispensed in each mold to form nine 20 × 10 × 0.2 mm cantilevers per batch. Within 1 min after casting, polydimethylsiloxane (PDMS) stamps were placed on the still‐liquid hydrogel mixture. The PDMS stamps were fabricated as previously described,^[^ ^6^ ^]^ and presented line features with 10 µm wide, 5 µm deep grooves, and 5 µm wide ridges. After 2 h, the PDMS stamps were carefully removed, and the edges of the cantilevers were cut using a scalpel. Then, the PLA molds were lifted and removed, while the hydrogel cantilevers remained on the glass slide. To detach the cantilevers, They were soaked in distilled water for 5 min and then were carefully scraped from the glass using a razor blade. The hydrogel cantilevers were stored at 2 °C in a parafilm sealed petri dish. The Mgel solution preparation is described in Table 1.

### Characterization of Mgels

Profilometer: The profilometer imaging was conducted using Olympus LEXT 4000 optical profilometer. The Mgel was imaged to demonstrate the microgrooves created by patterning it with a PDMS stamp, as previously described. Collected data were processed, using Matlab 2020b (The MathWorks Inc., Natick, MA, USA).

Instron: Mgel solutions with different MNP concentrations were prepared as described above and were cast into cylinder shapes (10 mm diameter, 4.5 mm height). Stress–strain curves were derived using an Instron 5944 (Norwood, MA, USA) in uniaxial unconfined compression mode. The height variation was taken into account. The elastic modulus was determined by a linear fit of the linear‐elastic deformation in the 15–30% range.

Rheology: Mgel samples were prepared as described above and were cast into cylinder shapes (20 mm diameter, 1 mm height), by casting 0.628 mL of Mgel solution into 3D‐printed PLA molds. Frequency sweeps were performed, using a rheometer (Discovery Hybrid‐Rheometer 3 (DHR‐3), TA Instruments, USA) to extract *G*′ and *G* ^″^.^[^ ^48^ ^]^

### Engineering CM‐Tissues on the Surface of Mgel Cantilevers: Harvesting and Seeding Neonatal Rat Ventricular Myocytes

Neonatal rat ventricular myocytes were isolated according to Tel Aviv University's ethical use protocols from 1‐ to 3‐day‐old Sprague‐Dawley rats as previously reported.^[^ ^49^ ^]^ Cells were isolated using six cycles (37 °C, 30 min, each) of enzymatic digestion with collagenase type II (95 U mL^−1^) and pancreatin (0.6 mg mL^−1^) in DMEM (Biological Industries, Beit‐Haemek, Israel). After each round of digestion, cells were centrifuged (600 × *g*, 5 min) and resuspended in M‐199 culture medium supplemented with 0.6 × 10^−3^ m CuSO_4_·5H_2_O, 0.5 × 10^−3^ m ZnSO_4_·7H_2_O, 1.5 × 10^−3^ m vitamin B12, 500 U mL^−1^ penicillin, and 100 mg mL^−1^ streptomycin, and 0.5% fetal bovine serum (FBS). To enrich the cardiomyocyte population, cells were suspended in culture medium with 5% FBS in flasks and were preplated twice for 45 min. Cell number and viability were determined by a hemocytometer and trypan blue exclusion assay.

Cell seeding on cantilevers was performed in custom‐made, PDMS‐cast seeding wells that were fitted to the cantilevers’ shape (Figure S2A, Supporting Information). These wells were designed to seed only half of the cantilever's area, by using a divider (Figure S2B, Supporting Information). The purpose of seeding only half the cantilever's area was to ensure that, in this experimental setup, the clamped‐down portion of the cantilever would not contain any cells, thereby preventing damage to the engineered cardiac tissue (Figure S2C,D, Supporting Information).

The seeding device and the 3D‐printed PLA (RaisePro, Raise Technologies, Inc.) dividers were sterilized in a 70% ethanol bath and transferred into a biological hood. The Mgel cantilevers were sterilized at ≈4000 µW s cm^−2^ for 2 min on each side. Then each Mgel cantilever was placed in one seeding well and a divider was placed in the middle. 0.4 mL of M199 media was then added with 120 000 cardiomyocytes to the patterned side of the Mgel cantilever, and M199 media without cells was dispensed on the unpatterned side to create equilibrium. After 6 h, the cantilevers were washed with PBS three times and transferred into 12‐well plates. 2 mL of fresh M199 media was added t each well. The medium was changed every 24 h. The cantilevers were used for experiments on day 5, where contractility of CM was observed (Movie S3, Supporting Information).

### Viability Assay

CMs were seeded on Mgels (with MNP concentrations of 0, 15%, 30%, and 50%) at 2.5 × 10^5^ cells per well in a 12‐well plate. Cells were cultured for 5 days prior to the assay, with medium change every two days. For assessing viability (live/dead assay), the culture medium was removed, and cells were stained with 5 µg mL^−1^ Hoescht 33342 (Thermo‐Fisher, USA) and 40 µg mL^−1^ propidium iodide (Sigma‐Aldrich, USA) in M199 medium for 30 min. Next, the staining solution was removed and replaced with fresh, warm Tyrode's buffer (37 °C). Cells were visualized with an IX83 Olympus fluorescent microscope (Olympus, Japan), at UPLFLN20XPH ×20 objective, NA 0.5, using DAPI and cy3 filters; 9–18 fields were taken for each condition. The number of nuclei carrying Hoechst and EThD‐1 fluorescent signals was determined with a designated ImageJ macro and percentage of live cells was calculated.^[^ ^25^ ^]^

### Immunostaining

Cardiomyocytes were seeded on patterned Mgels (with MNP concentrations of 0, 15%, 30%, and 50%) at 2.5 × 10^5^ cells per well in a 12‐well plate. After 5 culture days, cells were fixed in 4% paraformaldehyde solution (Thermo fisher, USA), washed with PBS, and permeabilized with 0.1% triton X‐100 (Sigma‐Aldrich, USA) in ddH_2_O. Next, samples were blocked with 10% normal goat serum (Jackson Laboratory) in PBS containing 0.1% bovine serum albumin (BSA; Sigma‐Aldrich). Samples were then incubated overnight at room temperature in a humidified chamber with either mouse anti α‐actinin (Sigma‐Aldrich, USA) at 1:200 in PBS+0.1% BSA or rabbit anti connexin 43 (Abcam) at 1:400 in PBS+0.1% BSA. Alexa goat anti‐mouse 594 and goat anti‐rabbit 488 (Thermo fisher, USA), diluted at 1:500, were used as secondary antibodies and incubated for 1 h. Samples were washed with PBS and mounted with DAPI Fluoromount G (SouthernBiotech, USA). Image acquisition was performed on an Olympus FV3000 confocal microscope (Olympus, Japan), with a UPLXAPO ×60 objective/1.42 NA, equipped with FLUOVIEW acquisition and analysis software.

### Calcium Imaging

Cardiomyocytes that had been seeded on Mgels (with MNP concentrations of 0, 15%, 30%, and 50%) for 5 days were treated with 4 µm Oregon BAMPTA‐1 AM (Thermo fisher, USA) with 0.02% Pluronic F127 in DMSO (Thermo Fisher, USA). Cells were incubated for 30 min prior to calcium imaging with Olympus FV3000 confocal microscope (Olympus, Japan), with a UPLXAPO ×20 objective/NA0.7. Images were acquired at a frame rate of 250 ms.

### DTL Setup: Platform Overview

The DTL setup, as depicted in Figure S4 of the Supporting Information, enables external control the deflection of the Mgel cantilever, as well as electrical stimulation of the cardiac tissues and recording of the cantilever's motion.

In this setup, a Mgel cantilever was placed inside a clear (47 mm × 47 mm × 30 mm) PMMA chamber filled with phosphate buffered saline (PBS), so that it was horizontally positioned 8 mm above the bottom of the inside of the chamber. The walls of the chamber were 1 mm thick. The Mgel cantilever was clamped, as illustrated in Figures 1D and 5A, between the spacers separating it from the bottom of the chamber and a clamp that pressed it from the top, against the spacers. The clamp was tightly attached to the chamber's walls to be held in place.

As discussed throughout this paper, the basic idea of the setup is to exert force on the Mgel cantilever—generating preload on the tissues plated on the cantilevers—by exposing the cantilever to a magnetic field. To control the magnetic field, permanent magnets attached to the end of a linear motor (LinMot PS01‐23×160‐R) were used. The linear motor was controlled by a corresponding C1100 controller from the same company. The controller was connected to a personal computer via a USB to RS‐485 converter adapter, and the computer was connected to a National‐Instrument USB‐6003 Data Acquisition Card (DAC). The analog output of the DAC provided voltages in the range of ±10 V and a current of 3 mA.

To electrically stimulate the cells plated on the cantilever, toward inducing contraction, a custom‐made power amplifier that amplifies voltages up to ±120 V at a current of up to 100 mA was used. The amplifier's output voltage was connected to a pair of square platinum electrodes (30 mm × 35 mm) that formed the stimulating electric field around the cells on the cantilever. To synchronize the electric field with the magnetic field, the digital output from the motor controller was used as a trigger for the analog voltage output. The control software, written in Visual Studio, allowed t control the various system parameters and define the waveforms of the electric and magnetic fields applied on the Mgel cantilevers, as well as the phase between the electric field and the magnetic field (Figure S5E, Supporting Information).

### Image Analysis

A high‐speed camera (Chronos 1.4, Kron Technologies, Canada) was placed close to the DTL chamber so that the cantilever could be filmed from the side (Figure S4B, Supporting Information). The cantilevers were filmed with a frame rate of 240 fps. The recorded videos were saved as .mp4 files and then converted to .avi. Videos were further processed and converted to binary sequences to allow edge detection frame by frame. From the binary images, the cantilever's shape was fitted to a second order polynom, which was further used to derive the curvature and *x* and *y* positioning of the cantilever tip for each frame, using Matlab.

### Simulations

3D finite‐element‐method simulations were used to examine the mechanical behavior of the Mgel cantilevers in the presence of an externally applied magnetic field. Simulation software with the Magnetic Fields, No Currents module was used, alongside the Solid Mechanics module, to simulate the magnetic fields and the deformation of the gel under their influence. Multiple configurations of stacked magnets, similar to their arrangements in the experimental setup, were simulated. The gel, modeled as a linear elastic material with the properties of collagen, was constrained such that half of it was fixed by the apparatus, while the other half was free to move. The deformation was obtained through a body load derived from the interaction of the static magnetic fields due to the external magnets with the magnetite nanoparticles embedded in the gel, assumed to be uniformly and isotropically dispersed. A stationary solver was used to calculate the steady state for each configuration (position of the external magnet). In addition, the electrostatics (es) module was used to simulate the distribution of the electric potential created by the voltage applied to the two electrodes in the chamber. For simplicity—and given that no time‐dependent dynamics were simulated, and that any significant deformation of the gel was not experimentally observed in the configuration of the lowest magnetic force—gravity and buoyancy were neglected.

### Stimulation Protocols for Experiments with CM‐Tissue

In the experiments, the magnetic field was generated and electric stimulation was initiated as illustrated in Figure S5E of the Supporting Information. Specifically, for the magnetic field, the bottom motor arm was programmed to move up to a defined position relative to the bottom of the DTL chamber, to induce an initial downward deflection of the cantilever for 500 ms prior to electrical stimulation (preload). Electrical field stimulation (Figure 5D) was induced using a 5 ms monophasic rectangular wave with an intensity of 50% above the voltage that produced maximal contraction of the cantilever and with astimulation frequency of 0.5 Hz.

### Force–Length Loops as an Equivalent Model to Pressure–Volume Loops

To measure contractility in the DTL, a model based on FL relations that are suggested to be equivalent to the PVR model used for measuring contractility in vivo was proposed. This model describes the relationship between the force generated by the cell, denoted *F*_cell_, and the length of the cell layer *L*.

The model includes the following assumptions.

- The Mgel cantilever is referred to as a deflecting beam.
- For a given deformation the beam conforms to the assumption of constant curvature equal to the average curvature, represented by

$c=\bar{c}=\frac{2W}{L_{0}^{2}}$, where *c* is the curvature (Bend downward to be positive), $\bar{c}$ is the average curvature, *W* is the deflection of the beam's end (downwards as positive), and *L*_0_ is the initial length of beam.

- All changes in kinetic energy are negligible (quasi‐static analysis).
- Small deformation hypothesis.
- Assuming the same drag coefficient everywhere on the beam.
- The beam's width and height are denoted as *b* and *h*, respectively (Figure S6A, Supporting Information).
- *X* describes the coordinate along the beam, where *x* ∈ [0,  *L*_0_], and *w*(*x*) is the beams deflection in a given point, where *w*(*L*_0_) = *W*. *w* is given by $w\left(x\right)=\frac{Wx^{2}}{L_{0}^{2}}$. The energy equation can be expressed as
(1) $$\bar{E}\mathrm{Ic}\dot{c}\;L_{0}=\underset{0}{\int^{L_{0}}}p_{m}\dot{w}dx+\underset{0}{\int^{L_{0}}}p_{d}\dot{w}dx-F_{\mathrm{cell}}\dot{L}$$

where the left‐hand side of the equation shows the rate of change of the beam's strain energy. $\bar{E}=\frac{E}{1-v^{2}}$ and $I=\frac{1}{12}\;bh^{3}$ are the equivalent modulus and moment of inertia, respectively, where *E* and *v* are, respectively, the Elastic modulus and the Poisson ratio corresponding to the substrate layer. The first term on the right‐hand side of Equation (1) is the time derivative (power) of the work done by the magnetic field force while *p*_m_ is the magnetic field force density. The second term on the right‐hand side of the equation is the time derivative (power) of the work done by the drag force on the cantilever beam, and $p_{d}\approx -\frac{3\mu \pi (3b+2L_{0})}{5L_{0}}\dot{w}$ is the drag force density, where *µ* is the viscosity of the fluid.^[^ ^50^ ^]^

The length of the cell layer can be expressed as

(2) $$L=\frac{L_{0}ch}{2}=\frac{Wh}{L_{0}}$$

and the tension force generated by the cells is obtained as

(3) $$F_{\mathrm{cell}}=-\frac{Ebh^{2}W}{3\left(1-v^{2}\right)L_{0}^{2}}+\frac{p_{m}L_{0}^{2}}{3h}-\frac{3\mu \pi \left(3b+2L_{0}\right)\dot{W}L_{0}}{25h}$$

The magnetic field force density relates to the distance between the magnet and the plane of the cantilever, and it can be determined by measuring the deformation of the cantilever beam in the presence of magnetic field forces only

(4) $$P_{m}\left(d\right)=\frac{2W_{m}Ebh^{3}}{3\left(1-v^{2}\right)L_{0}^{4}}$$

In this equation, *W*_m_ is the deflection of the endpoint of the beam (without the cell layer), with only magnetic field forces, and *d* is the distance between the magnet and the plane of the cantilever.

The model's parameters are given in Table S1 of the Supporting Information.

### In Vivo Pressure‐Volume Loops

All animal experiments were conducted according to the institutional animal ethical committee guidelines, which conform to the Guide for the Care and Use of Laboratory Animals published by the US National Research Council (Eighth edition 2011). Sprague‐Dawley 370 g male rat (Envigo Ltd, Jerusalem, Israel) was maintained at a constant temperature and relative humidity under a regular light/dark schedule (12:12), fed with normal rodent diet and tap water ad libitum.

### Measurements of Digoxin Effect on Cardiac Hemodynamics

For direct cardiac function evaluation, hemodynamic measurements were obtained by the Millar pressure–volume system (MPVS‐300, Millar Instruments, Houston, TX, USA). For the measurement, rats were placed on controlled heating pads and injected with a combination of 87 mg kg^−1^ ketamine and 13 mg kg^−1^ xylazine (I.M). Next, the right carotid was exposed and ligated distally, the artery was clamped and incised, and a 2‐Fr Mikro‐Tip catheter (SPR‐838, Millar Instruments, Houston, TX, USA) was advanced through the artery into the LV under pressure control; a ligature was then tightened around the catheter to avoid blood loss.^[^ ^51^ ^]^

Concomitantly, the left jugular vein was isolated traumatically, ligated distally, and clamped proximally. The vein was incised and a PE‐50 tube was inserted into the vein, small (<100 µL) of blood was withdrawn and the same volume of normal saline (0.9% NaCl) was injected into the vein, to establish a venous line to administer the drug.

### Drug Preparation

Digoxin was dissolved in DMSO to a concentration of 10 mm, and further diluted in normal saline to a dosage of 0.5 mg kg^−1^.

Data acquisition: After signal stabilization for 8 min, signals were continuously sampled at a sampling rate of 1000 samples per second by the MPVS‐300 system, recorded, and displayed on a personal computer by the PowerLab System and Chart5 software (AD Instruments, Colorado Springs, CO, USA) for 10–15 min.

Digoxin treatment was given, through the venous line, after 15 min of baseline recording.

Every few minutes, throughout the sampling (baseline and under digoxin), an occlusion maneuver was conducted to assess the end‐systolic pressure–volume relationship (ESPVR) and the cardiac contractility.

At the end of each experiment, several boluses of 100 µL of hypertonic (30%) saline were injected intravenously, and from the averaged shift of PV relations, parallel conductance volume (*V*_p_) was calculated by the software and used for the correction of the cardiac mass volume. Thereafter, the catheter was withdrawn, and the animal was sacrificed.

### Data Analyses

The Millar PV system simultaneously and continuously measures LV pressure and volume from the intact beating heart, producing characteristic PV loop readings. A variety of cardiovascular parameters, such as heart rate, cardiac output, stroke volume, ejection fraction, stroke work, d*P*/d*t*_max_, and d*P*/d*t*_min_ are derived from each PV loop. Additionally, occlusion maneuvers were analyzed for ESPVR and contractility assessments.

### Ethical Statement

All experiments were approved by the local veterinary authority and the animal ethics committee of Tel Aviv University (Ethical Approval Number: 01‐19‐079) and were performed in accordance with Israeli law. All efforts were made to minimize animal suffering and to reduce the number of animals used.

## Conflict of Interest

The authors declare no conflict of interest.

## Supporting information

Supporting Information

Supplemental Movie 1

Supplemental Movie 2

Supplemental Movie 3

Supplemental Movie 4

Supplemental Movie 5

## Data Availability

The data that support the findings of this study are available in the Supporting Information of this article.
