# Supplementary material for: A Platform for Assessing Cellular Contractile Function Based on Magnetic Manipulation of Magnetoresponsive Hydrogel Films
Source: Adv Sci (Weinh). 2023 Jul 23;10(27):2207498. doi: 10.1002/advs.202207498 (PMC10520681; doi:10.1002/advs.202207498)
Supplement: Supplementary file 1 — Supporting Information [file ADVS-10-2207498-s004.pdf]

ADVANCED SCIENCE
Open Access

Supporting Information

for *Adv. Sci.*, DOI 10.1002/advs.202207498

A Platform for Assessing Cellular Contractile Function Based on Magnetic Manipulation of Magnetoresponsive Hydrogel Films

Moran Yadid, Mario Hagel, Megan Beldjilali Labro, Baptiste Le Roi, Carina Flaxer, Eli Flaxer, A. Ronny Barnea, Shai Tejman-Yarden, Eric Silberman, Xin Li, Rossana Rauti, Yael Leichtmann-Bardoogo, Hongyan Yuan and Ben M. Maoz**

Supporting Information for:

A Platform for Assessing Cellular Contractile Function Based on Magnetic Manipulation of Magneto-Responsive Hydrogel Films

Moran Yadid[1, 2†#], Mario Hagel[3†], Megan Beldjilali Labro[3], Baptiste Le-Roi[3], Carina Flaxer[3], Eli Flaxer[4], A. Ronny Barnea[3], Shai Tejman-Yarden[5,6,7], Eric Silberman[2], Xin Li[8], Rosana Rauti[9], Yael Leichtmann-Bardoogo[3], Hongyan Yuan[8], Ben M. Maoz[3,10,11#]

[1]The Azrieli Faculty of Medicine, Bar Ilan University, 8 Henrietta Szold St., Safed, 1311502, Israel

[2]The Shmunis School of Biomedicine and Cancer Research, Tel Aviv University, Tel Aviv, 69978, Israel

[3]Department of Biomedical Engineering, Tel Aviv University, Tel Aviv, 69978, Israel

[4]AFEKA - Tel-Aviv Academic College of Engineering, 69107 Tel-Aviv, Israel.

[5] The Edmond J. Safra International Congenital Heart Center, Sheba Medical Center, Ramat Gan, Israel

[6] The Engineering Medical Research Lab, Sheba Medical Center. Ramat Gan, Israel

[7]The Sackler School of Medicine, Tel Aviv University, Tel Aviv, Israel

[8]Shenzhen Key Laboratory of Soft Mechanics & Smart Manufacturing, Department of Mechanics and Aerospace Engineering, Southern University of Science and Technology, Shenzhen, 518055, China

[9] Department of Biomolecular Sciences, University of Urbino Carlo Bo, Urbino, 61029, Italy

[10] Sagol School of Neuroscience, Tel Aviv University, Tel Aviv, 69978, Israel

[11] The Center for Nanoscience and Nanotechnology, Tel Aviv University, Tel Aviv, 69978, Israel

Supplementary Figures:

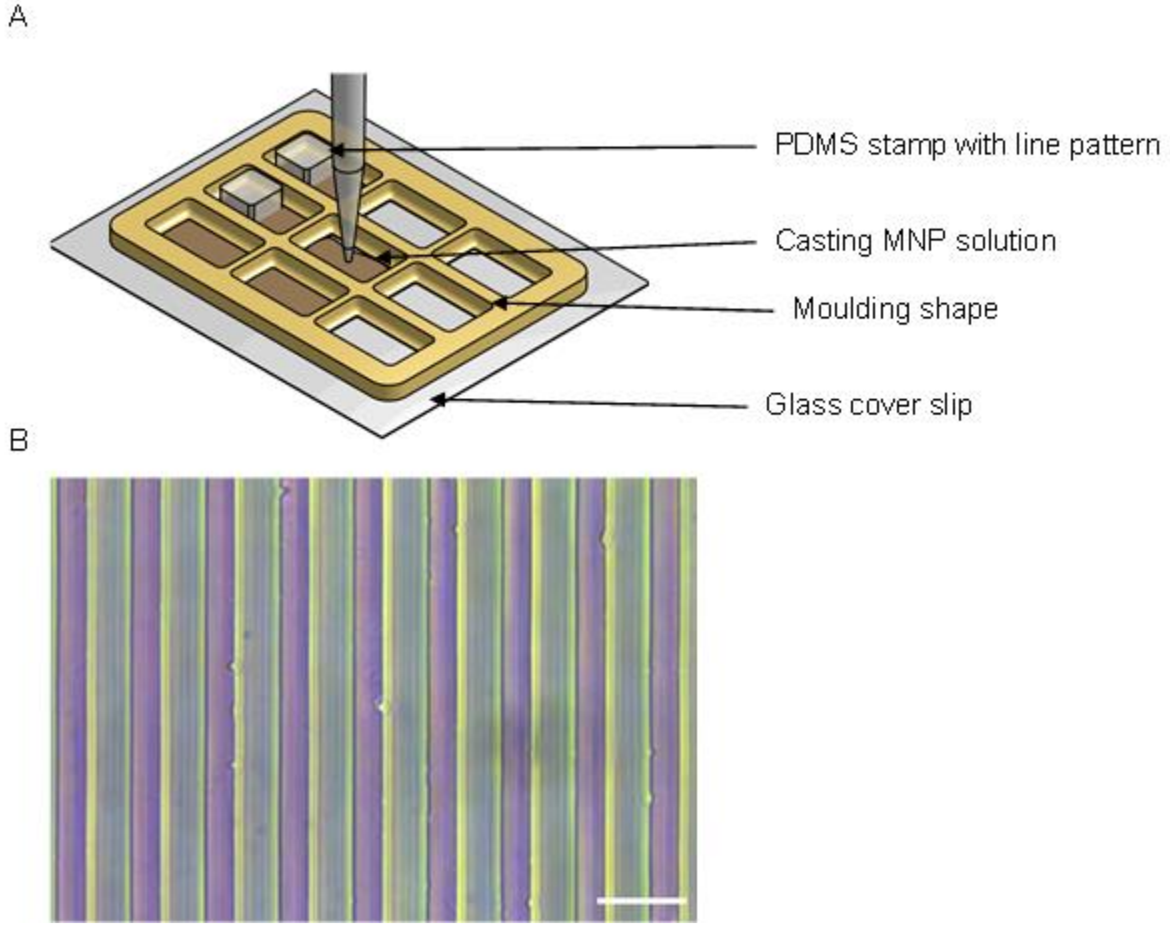

Figure S1. Casting gels into casting template. (A) Casting of the MNP solution into the molding shape with the PDMS stamp. **(B)** Microscope image of the linear patterned stamps. Scalebar 10µm.

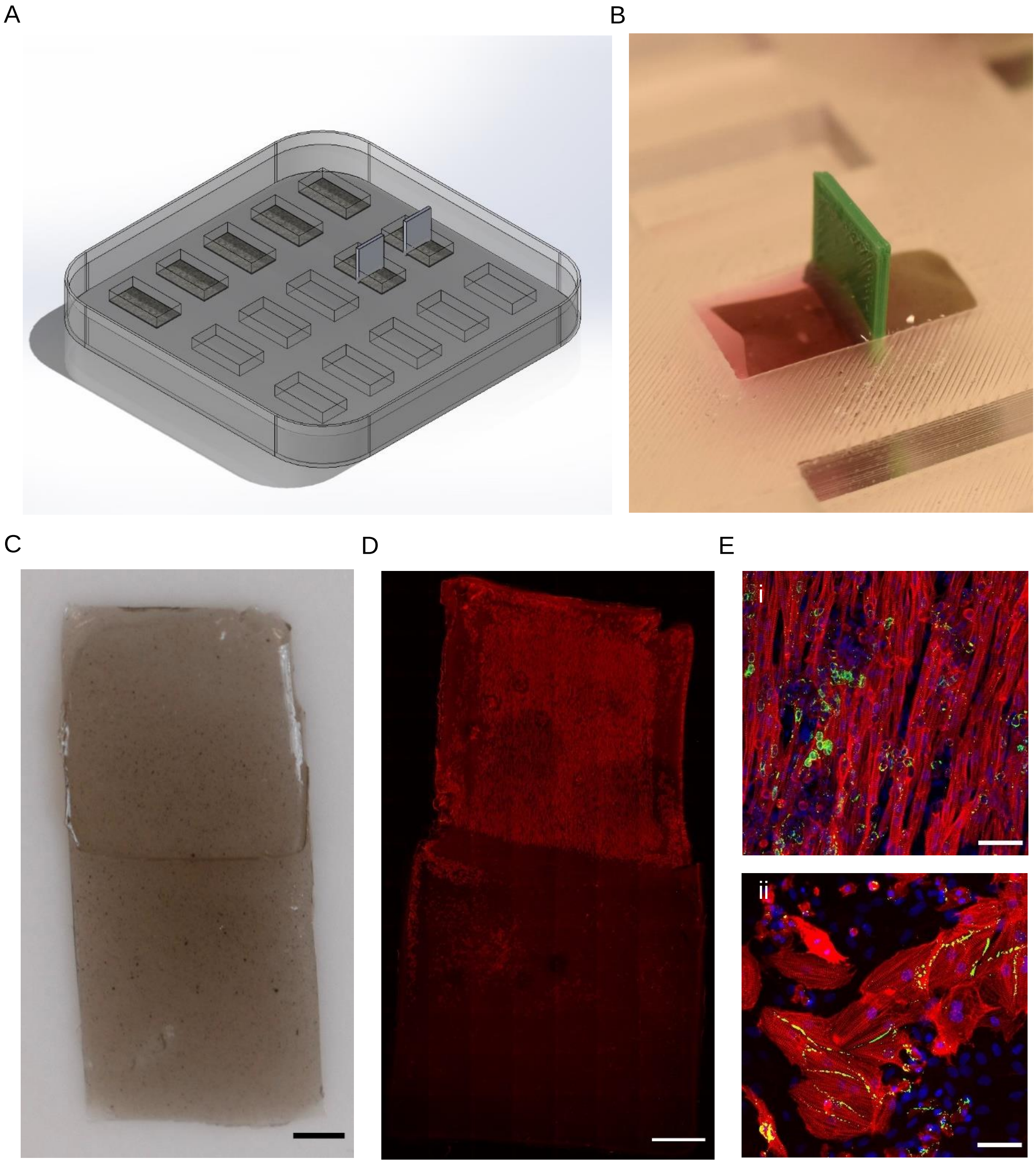

Figure S2. CM seeding. (A) The seeding device is a PDMS inlay for a square petri dish (120 mm × 120 mm). It was produced by 3D printing a PLA mould that is the mirror shape of the seeding device. The 3D printed mould was placed in the square petri dish, and a mixture 1:10 ratio of PDMS cross-linker was casted onto the mould. Then it was left in the desiccator for 1 hour to dissolve air bubbles and left untouched for 2 days. Afterwards the formed PDMS seeding well device was removed from the mould and placed in a clean square petri dish.
After sterilization it can be used for the seeding procedure, using 3D printed rectangular dividers that are used for reducing the effective seeding area. **(B)** One well with a cantilever and a divider

(3D printed), separating the CM-seeded side from the other, unseeded side. Left side with red liquid shows medium with cells and the right side medium without cells, whereby an equilibrium is created to prevent leakage of cell-containing media from the seeded side to the unseeded side. **(C)** Cantilever with 15% MNP. Scale bar: 2mm. **(D)** Cantilever with seeded and aligned cardiomyocytes in the upper micropatterned area and unpatterned lower area. Scale bar: 2mm.**(E)** Immunostaining overlay of CMs: DAPI (blue) – Nucleoli. Alpha-actinin (red) - sarcomeres. Connexin 43(green) – tight junctions. Scalebars: 10 µm. **(Ei)** CMs aligned on a patterned hydrogel. **(Eii)** CMs on an unaligned hydrogel.

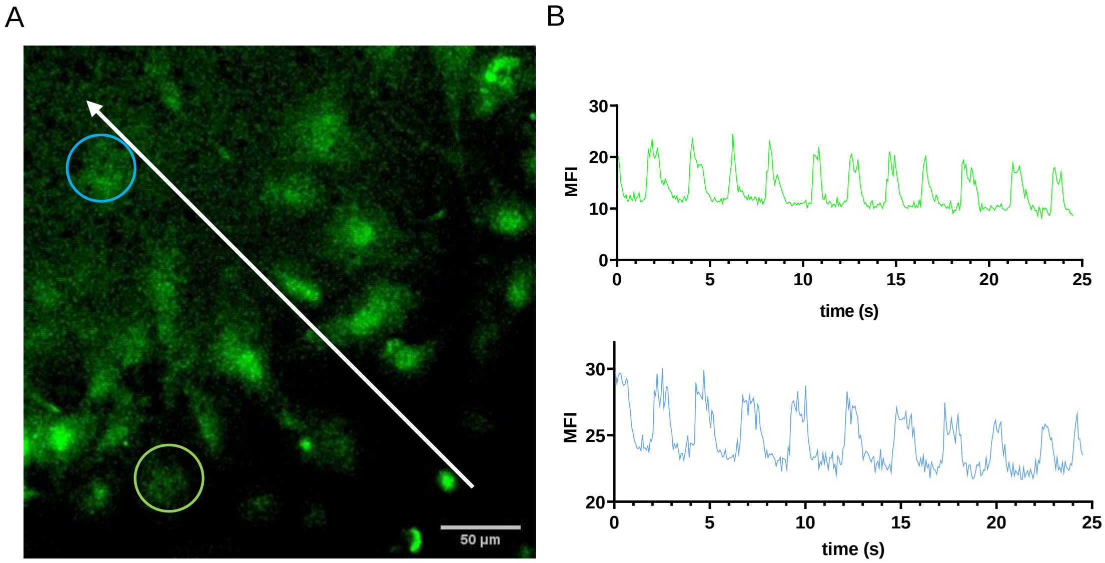

Figure S3. Calcium imaging. (A) Repetitive spontaneous Ca^{2+} events. The white arrow represents the direction of signal propagation. **(B)** The circles mark the areas that were plotted as mean fluorescence intensity (MFI) over the time (s). The peaks represent the measured calcium waves.

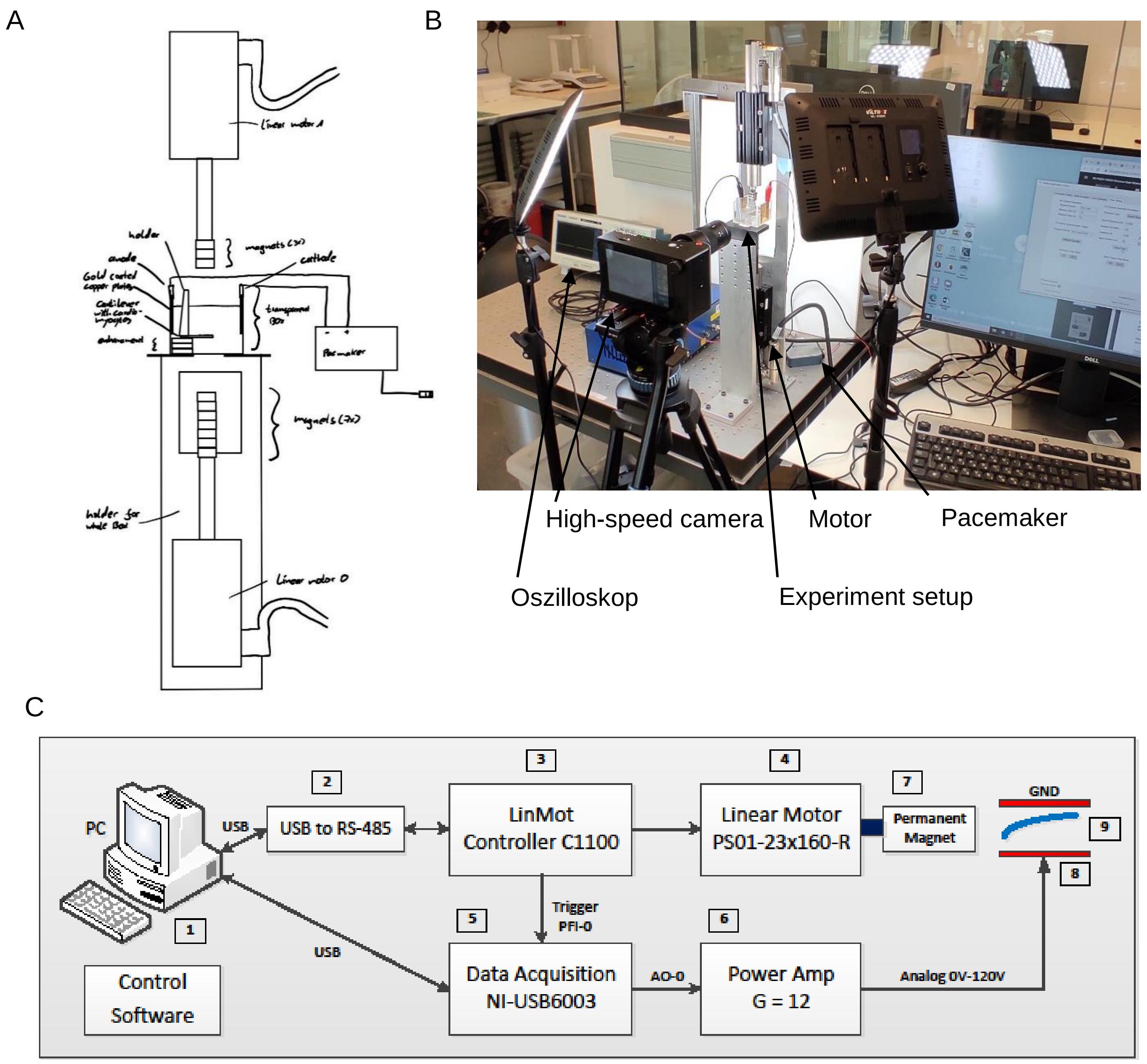

Figure S4. DTL platform and setup. (A) Schematic of the general setup. **(B)** Shows the general setup with high-speed camera, oscilloscope, motor and pacemaker. **(C)** Control system. On the cantilever [9], which contains magnetic particles, force is exerted by a change in the magnetic field around it. To control the magnetic field, we used a permanent magnet [7] attached to the end of a linear motor [4]. The LinMot PS01-23x160-R linear motor is controlled by a C1100 suitable controller from the same company [3]. The controller connects to the personal computer [1] through the USB to RS-485 converter adapter [2]. The computer is also connected to a National-Instrument USB-6003 Data Acquisition Card (DAC) [5]. The analogue output of the DAC provides voltages in the range of ±10 V and current of 3mA. We use a power amplifier [6] that amplifies the voltages up to ±120 V at a current of up to 100 mA. The voltages at the amplifier output are connected to electrodes [8] that form the electric field around the cells on the cantilever. To synchronize the electric field with the magnetic field, we use a digital output from the motor controller and use it as a trigger for the analogue voltage output.

The control software, written in Visual Studio, allows us to control all the parameters in the system. The software can determine the position of the magnet in space depending on time (frequency, amplitude, and spatial waveform). In addition, the software determines the analogue waveform that controls the electric field, and the phase between the electric field and the magnetic field.

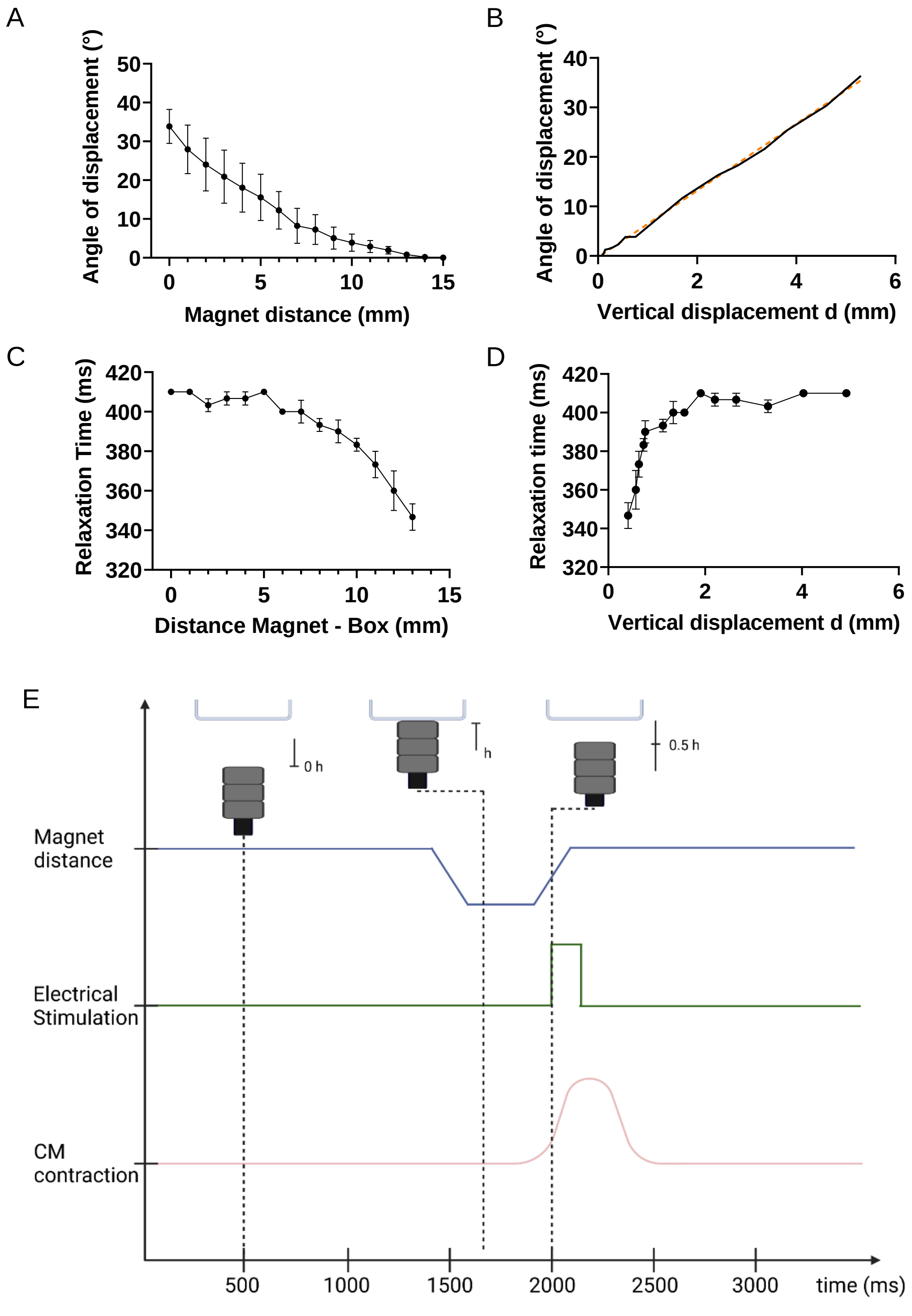

Figure S5. Characterization of cantilever's response to magnetic field. (A) The angle of the downward deflected cantilever as a function of the magnets' distance from the bottom of the DTL chamber. **(B)** The angle of the downward deflected cantilever as a function of the vertical displacement of the cantilever's tip for a given magnet position, demonstrates a linear relationship. **(C-D)** Cantilever's relaxation time is measured as the time it takes the cantilever to return to its initial position after retrieving the magnets back to their homing position (to their maximal distance from the cantilever), as a function of the distance of the magnets when close to the DTL box **(C)** and as a function of the corresponding vertical displacement of the tip **(D)**; **(E)** Schematic depiction of the sequence of events that induce preload, electrical stimulation, and the consecutive contraction of the myocardial tissue constructed on the cantilever.: First, the magnets are brought closer to the cantilever, to pre-stretch it, as apparent by the cantiliever's downward deflection, for 500ms. Then as the pre-stretch is released, when the motor arm with magnets is halfway down, back to its initial homing position, a 50ms electrical pulse is induced, leading to activation and mechanical contraction of the myocytes, apparent as the upward deflection of the cantilever.

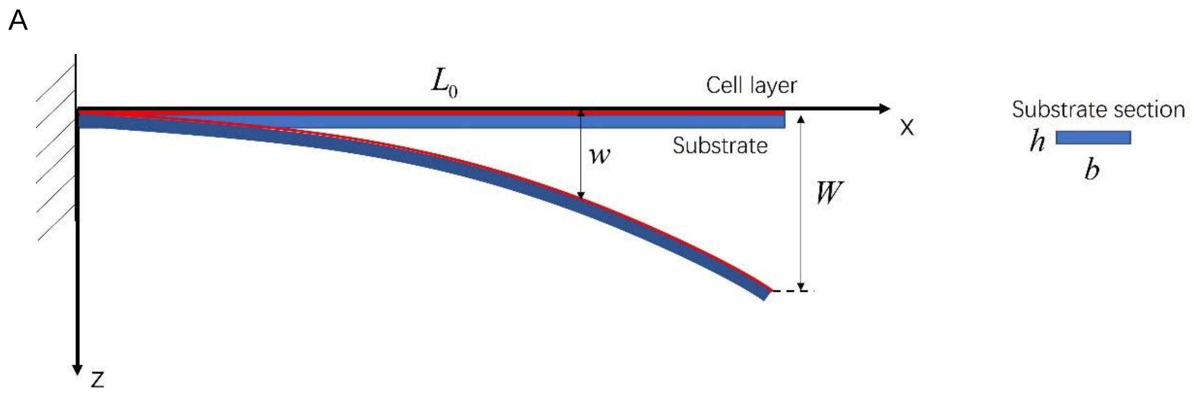

SI Figure 6. Schematic of cantilever deflection. (A) This schematic, depicting the bending beam model, was used to derive the mathematical model according to which the myocardial tension was calculated. The blue beam represents the Mgel film in the initial horizontal position along the x-axis and as it bends due to uniformly applied force, with the vertical distance W at the tip. On top of it, the red streak represents the cardiomyocyte layer, which is assumed to be very thin. On the right side, the cross-section can be seen, with height and width described by h and b.

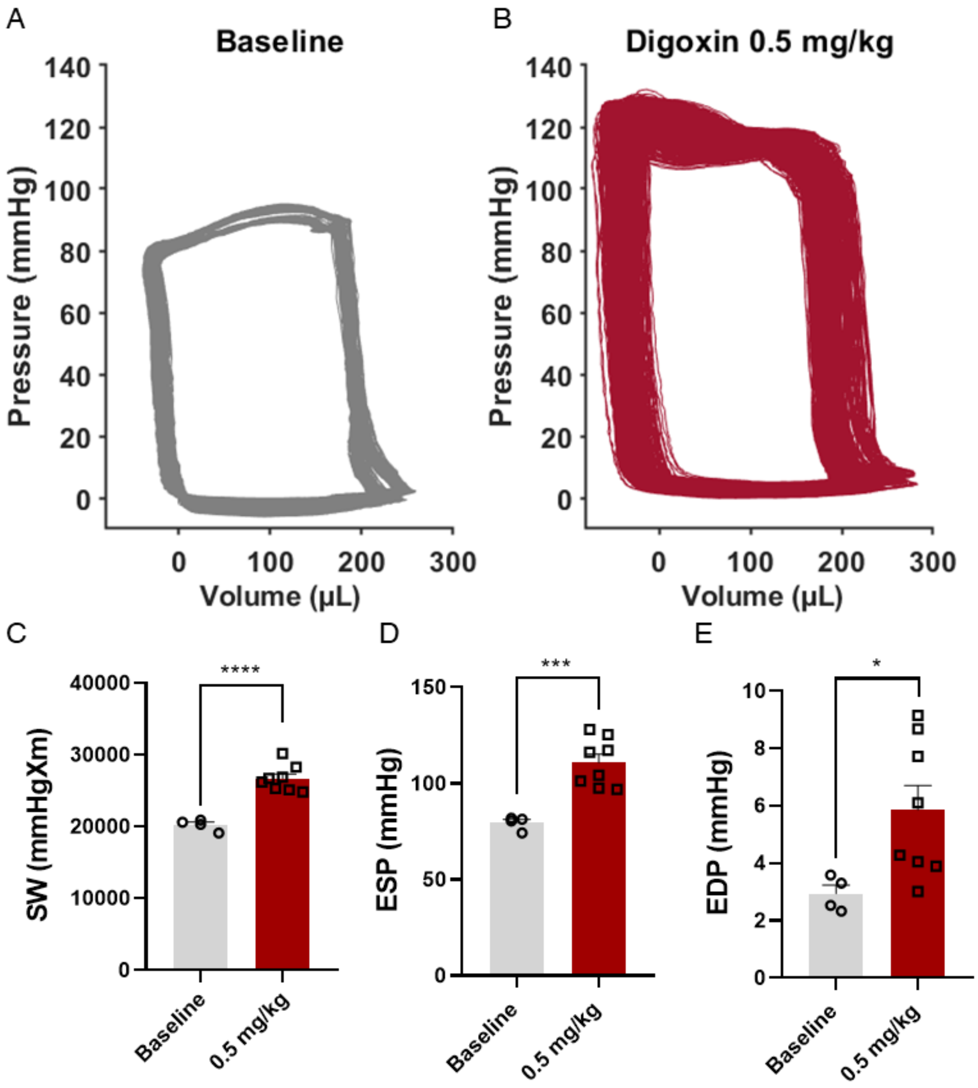

SI Figure 7. Derived Pressure-Volume Loop *in vivo* with and without Digoxin. (A) The baseline of the PV-Loop that was measured *in vivo* in a rat. **(B)** PV-Loop that was measured *in vivo* in a rat with added Digoxin dosage of 0.5 mg/kg, showing an increase in pressure. **(C)** Systolic Work (SW) was calculated before and after the induction of digoxin. **(D)** End-systolic pressure (ESP) for with and without Digoxin dosage, showed a statistically significant difference. **(E)** End-diastolic pressure (EDP) with and without Digoxin. * $p < 0.05$, ** $p < 0.01$, *** $p < 0.001$, **** $p < 0.0001$, ns $p \geq 0.05$.

Supplementary Movies:

SI Movie 1i - shows the response of the Mgel to magnetic field.
SI Movie 1ii - shows the response of the cardiomyocytes cultured on the Mgel to electric field.
SI Movie 1iii- shows the response of the cardiomyocytes and Mgel to electrical and magnetic stimulation and manipulation.
SI Movie 2 - Finite-element simulation which simulate the magnetic flux density at the plane of the cantilever
SI Movie 3 – Spontaneous contraction of cardiomyocytes that were cultured on the Mgel for 5 days

Supplementary Tables:

SI Table 1. Parameters, values & units. The table shows the parameters and the corresponding values used to derive the results, using Force – Length Loop relations.

**Parameter**		**Value**	**Unit**
E-modulus	E	50 000	Pa
Width Mgel	b	10	mm
Length free Mgel	L_0	10	mm
Height Mgel	h	0.2	mm
Poissons ratio	ν	0.5	-
Absolut viscosity	μ	0.001002	Pa*s

Mgel bending at tip without cellular layer W_m	Measured variable	mm
Mgel bending at tip with cellular layer W	Measured variable	mm
Time per frame Δt	0.00416 (1/240)	s
Magnetic applied force p_m	Measured variable	N/mm^2
